# Multidirectional Plyometric Training: Very Efficient Way to Improve Vertical Jump Performance, Change of Direction Performance and Dynamic Postural Control in Young Soccer Players

**DOI:** 10.3389/fphys.2019.01462

**Published:** 2019-12-09

**Authors:** Mohamed C. Jlid, Ghazi Racil, Jeremy Coquart, Thierry Paillard, Gian Nicola Bisciotti, Karim Chamari

**Affiliations:** ^1^Higher Institute of Sport and Physical Education of Ksar Saïd, University of Manouba, Tunis, Tunisia; ^2^Laboratoire CETAPS, Université de Rouen, Rouen, France; ^3^EA4445 Laboratoire Mouvement, Equilibre, Performance, Santé (MEPS), Tarbes, France; ^4^ASPETAR, Qatar Orthopaedic and Sports Medicine Hospital, Doha, Qatar

**Keywords:** muscle power, stretch-shortening-cycle, agility, balance, adolescents

## Abstract

The aim of the study was to assess the effects of multidirectional plyometric training (MPT) on vertical jump height, change of direction performance (CODP), and dynamic postural control (DPC) in young soccer players. Twenty-eight young male soccer players were randomly assigned to an experimental group (EG, *n* = 14; age: 11.8 ± 0.4 years) and a control group (CG, *n* = 14; age: 11.6 ± 0.5 years). The EG introduced 8-week MPT, two days per week into their in-season training, while CG continued training without change. Measurements of vertical jump height, CODP, and DPC were completed at the beginning and end of the 8-week MPT. A significant group × time interaction was observed for Squat-Jump (*p* < 0.05), for Counter-Movement Jump (*p* < 0.05), and for CODP test (*p* < 0.05). In addition, a significant group × time interaction was observed for DPC in seven axes for the dominant- (anterior, lateral, postero-lateral, posterior, postero-medial, medial, and antero-medial; *p* < 0.05 for all) and in seven axes for the non-dominant- (anterior, antero-lateral, lateral, posterior, postero-medial, medial and antero-medial; *p* < 0.05 for all) legs. The rest of the axes of both legs did not show any significant group × time interaction (*p* > 0.05). In conclusion, incorporating MPT into the in-season regimen of young male soccer players improved performance of various indices related to soccer activity (i.e., vertical jump height, CODP, and DPC). MPT has the potential to be appealing to coaches, as it requires little time while yielding valuable results in the physical preparation of young soccer players.

## Introduction

Soccer is an intermittent sport in which the capacity of the player to perform actions such as sprinting, jumping, kicking and changing direction have a major influence on match performance (Stolen et al., 2005). Research has characterized the intermittent activity pattern of youth soccer players. Rebelo et al. (2014) reported a match total distance covered of 6311 ± 948 m, of which 12% were performed at high-intensity activities. Likewise, Castagna et al. (2003) showed that 9% of the total time played (3789 ± 109 s) were performed at high-intensity activity with similar duration (11%) spent standing still.

Many soccer-specific movements are characterized by high-velocity concentric and eccentric muscular contractions, involving muscular stretch-shortening cycle (SSC). In that regard, plyometric training (PT) is known to improve the ability of soccer players to cope with the game demands. More specifically, PT can develop the ability of soccer players improving their neuromuscular control by promoting anticipatory postural adjustments (Gantchev and Dimitrova, 1996; Asadi et al., 2015). Indeed, balance and stability challenges during PT can result in proactive and/or feed-forward adjustments that would adjust appropriate muscles contractions before pitch-contact/landing (Marigold and Patla, 2002; Paillard et al., 2005). Furthermore, PT seems to result in improved sensitivity of afferent feedback pathway during exercise (Borghuis et al., 2008). Bedoya et al. (2015) recently suggested that the observed gains in performance could reflect various neuromuscular adaptations, such as an increased neural drive, improved inter-muscular coordination, changes in muscle size and architecture, and/or changes in single-fiber mechanics, as well as changes in muscle-tendon mechanical-stiffness (Markovic and Mikulic, 2010). Therefore, all these improvements could increase the performance and also potentially minimize the risk of injuries in soccer players (Chimera et al., 2004). Moreover, PT is attractive to soccer coaches, because it requires little space or equipment, and uses short periods from the training sessions’ time (Ramirez-Campillo et al., 2014).

PT can take the form of vertical or horizontal exercises, or a combination of both. The SSC contributes less to horizontal than to vertical jumping performance, because a vertical loading of the musculo-tendinous unit accumulates greater elastic energy during movement the excentric phase (Kawamori et al., 2013). Ramirez-Campillo et al. (2015) showed that a combination of vertical and horizontal jumping yielded greater gains for both strength and balance of the players than vertical or horizontal jumping performed separately.

The soccer is multidirectional sport (Taylor et al., 2017) and consequently the adequate physical preparation must meet such a characteristic. The study of Geoff et al. (2016) in Badminton has shown that resistance and multidirectional PT among badminton players has improved their specific physical qualities in this multi-directional sport. Consequently, as soccer is also a multi-directional sport, one may hypothesize that the optimal preparation of soccer players should also include multidirectional exercises.

Although some authors have argued that PT is detrimental to young players, increasing the risk of injury and stunting growth, such issues are easily avoidable if an age-appropriate regimen is followed (Behm et al., 2008; Faigenbaum et al., 2009). Current guidelines for youth (Behm et al., 2008; Faigenbaum et al., 2009) require that PT be carried out on 2–3 non-consecutive days per week for 8–10 weeks, and that the volume of training should be relatively low (generally approximately 60 foot contacts per session, and increasing to no more than 120 foot contacts per session) (Ramirez-Campillo et al., 2013). In terms of efficacy, a recent study in young male soccer players (Chaabene and Negra, 2017) indicated that over 8 weeks of training, a high-volume program had no greater effects than a low-volume program on sprint time, change of direction performance (CODP), or jumping performances. The effectiveness of low-volume training, resulting as effective a high-volume plyometric program in young soccer players could be explained by the intermittent nature of young soccer players’ activity during the matches. Indeed, the total match time is characterized by 11% of standing time and 9% of high-intensity activity (Castagna et al., 2003). The relatively low amount of high-intensity activity and the capacity in young players to recover faster from intense exercise than adults (Hebestreit et al., 1993) could potentially explain the effectiveness of low-volume polymeric program in young soccer players.

An optimal training regimen for young soccer players should also enhance dynamic postural control (DPC) (Paillard et al., 2006), minimizing the risk of lower-extremity injuries (Zech et al., 2010) through an increased contraction force in the lower extremity muscles (Myer et al., 2006), and/or enhancement of proprioception and neuromuscular control (Hewett et al., 2002). The effects of multidirectional plyometric training (MPT) on the DPC of young soccer players are as yet unknown, but a favorable adaptation might be expected given that the increases in the lower-extremity muscle power can be associated with improvements in postural performance since there would be a relationship between very early rapid torque of the leg extensor muscles and performance postural in young subjects (Paillard, 2017b).

The vertical jump performance, CODP, and DPC are soccer specific physical qualities and their improvement might improve soccer performance in young soccer players (Meylan and Malatesta, 2009; Ramirez-Campillo et al., 2015; Negra et al., 2017). Therefore, the main objective of this research was to study the effects of 8-week in-season MPT on the vertical jump performance, CODP, and DPC of young soccer players. We hypothesized that MPT would enhance these indices of physical performance abilities in young soccer players.

## Materials and Methods

### Participants

All procedures were approved by the Manouba University Institutional Review Committee for the ethical use of human participants and were conducted in accordance with the Declaration of Helsinki. Written informed consent was obtained from all participants and their parent after they had received both verbal and written explanations of the experimental protocol and its potential risks and benefits. All participants were ensured that they could withdraw from the trial at any time without any penalty.

The participants were 28 male soccer players from a soccer academy (all playing positions except goalkeeper) (Table 1). They were randomly assigned to an experimental group (EG; *n* = 14) and a control group (CG; *n* = 14). None reported any recent history of hip, knee or ankle injury, or other pathological conditions affecting their lower limbs.

**TABLE 1:** Participants characteristics before the intervention program (mean ± SD).

**Variable**	**Control group**	**Experimental group**
Age (yr)	11.6 ± 0.5	11.8 ± 0.4
Body height (m)	1.42 ± 0.04	1.43 ± 0.10
Body mass (kg)	34.2 ± 3.6	36.5 ± 5.1
Leg length (m)	0.85 ± 0.03	0.86 ± 0.10
Body mass index (kg/m^2^)	16.8 ± 1.2	17.8 ± 1.8
Time to predicted PHV (yr)	−2.0 ± 0.4	−1.9 ± 0.3
Soccer experience (yr)	3.8 ± 0.4	3.6 ± 0.5

*No inter-group differences are statistically significant (*p* > 0.05). PHV, peak height velocity.*

### Experimental Design

The study was performed over an 8-week period during March and April. The PT program was started at the 18th week of the training season starting in September and finishing in June. Two sessions for familiarization with the testing were held 2 weeks before the beginning of experimentation. Data were collected before modification of the regimen and after the EG had completed the 8-week period of MPT. Initial and final measurements were made at the same time of day (4 to 6 pm) (Chtourou et al., 2012) and under the same experimental conditions at least 3 days after the most recent competition, and 5 days after the last MPT session.

Testing was integrated into the weekly training schedule. A standardized warm-up, including progressive running and dynamic stretching exercises (Haddad et al., 2014), was performed before each testing session. Experimental tests were performed by the same investigator in a fixed order over 3 consecutive days. On day 1, anthropometric measurements were completed, followed by vertical jumping height tests (i.e., squat jump: SJ and countermovement jump: CMJ). Day 2 was devoted to assess the agility from COD obtained during *T*-test, and on day 3 participants undertook the DPC test to evaluate balance.

### Anthropometric Measurements

Height was measured using a graduated measuring rod (version 216, Seca^®^ Hamburg, Germany), and players were weighed with light clothes and barefoot, using an EKS^®^ Focus 9800 scale (Gislaved, Sweden). Body mass index (BMI) (kg/m^2^) was calculated as body mass (kg) divided by the square of height (m^2^). Moreover, an individual maturity index was calculated from peak height velocity. The method of predicting years from peak height velocity (PHV) was as follows: PHV = −7.999994 + [0.0036124 × age × height] (Moore et al., 2015). Finally, the dominant leg was determined according to the method of van Melick et al. (2017).

### Vertical Jump Height Assessments

Jump heights during SJ and CMJ were assessed using an infrared photocell connected to a digital computer (Optojump System, Microgate^®^, Bolzano, Italy). This allowed the measurement of contact time and flight time (t_*f*_) with a precision of 1/1000 s. The jump height (h) in meter was calculated as h = g × (t_*f*_)^2^/8 as indicated by Lehance et al. (2005). For the SJ, participants placed their hands on their hips in a semi-squat position, with the knees flexed to ∼90° (Ghoul et al., 2019). CMJ started from a standing position with the hands on the hips; at the verbal signal, a downward movement was made to a knee angle of ∼90°, and the participant then pushed upward as rapidly as possible (Ghoul et al., 2019). For both jumps, three trials were made, and the average used for analysis. For the SJ and CMJ, intraclass coefficients (ICCs) for 3 repeated trials before and after intervention period were 0.95 and 0.94; the 95% confidence interval (CI) were of 0.92–0.96 and also 0.92–0.96, respectively.

### Time to Complete Change of Direction Test

On the second test day, to assess the CODP, we have used a valid, reliable and sensitive test in soccer (Sporis et al., 2010): the *T*-test was administered as described by Semenick (1990). One cone was placed 9.14 m ahead of the starting cone and 2 further cones were placed 4.57 m on either side of the second cone. Times were recorded using an electronic timing gate (Photocells, Microgate^®^, Bolzano, Italy). The photoelectric cells were placed at 0.7 m height from the floor. Participants sprinted forward 9.14 m to the first cone, touching its tip with their right hand, next shuffled 4.57 m left to the second cone, touching it with their left hand, then shuffled 9.14 m right to the third cone, touching it with their right hand, next shuffled 4.57 m left to the middle cone, touching it with their left hand before finally running backward to the starting line of 2 m wide. Trials were deemed unsuccessful if participants failed to touch a designated cone, crossed their legs while shuffling or failed to face forward at all times. All participants performed familiarization trials before undertaking three definitive trials separated by one minute recovery intervals. The average of the three trials has been used for analysis. The ICC for 3 repeated trials before and after intervention period were 0.96 and with 95% CI ranging between 0.94 and 0.97, respectively.

### Dynamic Postural Control

**FIGURE 1:**
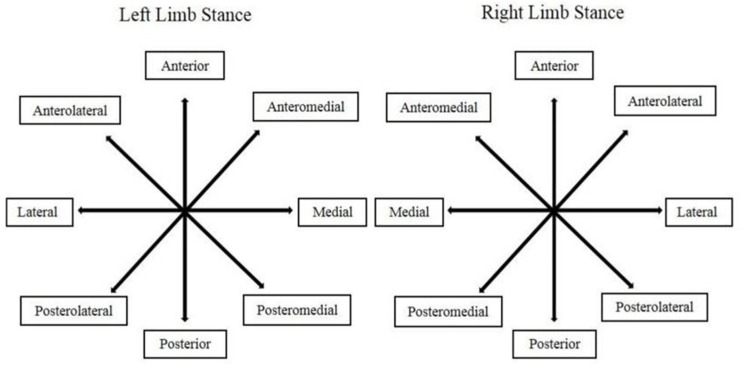
The 8 positions of the star excursion balance test are based on the stance limb.

During the third test day, the star excursion balance test assessed DPC. This unilateral balance test integrates a single-leg stance with maximum reach of the opposite leg (Hertel et al., 2000). Participants stood in the center of a grid, with 8 lines radiating at 45° increments from the center of the grid [antero-lateral (AL), anterior (A), antero-medial (AM), medial (M), postero-medial (PM), posterior (P), postero-lateral (PL), and lateral (L) (Figure 1)]. Reach distances were normalized by dividing each excursion distance by the participant’s leg length and multiplying the value obtained by 100. The average of the three trials has been used for analysis. ICCs for 3 repeated trials before and after intervention period of the 8 directions using dominant and non-dominant legs ranged from 0.90 to 0.94, with 95% CI ranging between 0.88 and 0.97, respectively.

### Multidirectional Plyometrics Training (MPT)

The EG integrated 20–25 min MPT sessions into their regular training sessions on every Tuesday and Thursday throughout the 8-week intervention period, whereas the controls continued with their standard soccer training in season (Tables 2, 3).

**TABLE 2:** Soccer training program during the week.

**Session 1 and Session 2**	**Session 3**
Warm up	Warm up
General	General
Specific	Specific
Training	Training
Training of fast footwork	Training of fast footwork
Technical skills and moves(easy/difficult)	Position games with ball(small/big)
Technical skills and moves(easy/difficult)	Tactical games with variousobjectives
Cool down exercises	Cool down exercises

**TABLE 3:** Multi-directional plyometric training program.

**Week**	**Exercises**	**Directions**	**Sets × repetitions per-session**	**Foot contacts per-session**
1	• Alternating jumps (right-left leg) forward throughout hoops	V-A	3 × 6	54
	• Alternating jumps lateral (right-left leg) throughout hoops	V-L	3 × 6	
	• Jumps with feet together and then separated throughout hoops	V-A-L	3 × 6	
2	• Alternating jumps (right-left leg) forward throughout hoops	V-A	4 × 6	72
	• Alternating jumps lateral (right-left leg) throughout hoops	V-L	4 × 6	
	• Jumps with feet together and then separated throughout hoops	V-A-L	4 × 6	
3	• Jumping, feet together throughout hoops	V-A	4 × 8	96
	• Alternating jumps lateral (right-left leg) throughout hoops	V-L	4 × 8	
	• Jumps with feet together and then separated throughout hoops	V-A-L	4 × 8	
4	• Jumping, feet together throughout hoops	V-A	4 × 8	104
	• Alternating jumps lateral (right-left leg) throughout hoops	V-L	4 × 9	
	• Jumps with feet together and then separated in hoops	V-A-L	4 × 9	
5	• Jumps forward between barriers (30 cm)	V-A	4 × 10	112
	• Lateral jumps over a bench (20 cm)	V-L	4 × 9	
	• Jumps with feet together and then separated throughout hoops	V-A-L	4 × 9	
6	• Jumps forward between barriers (30 cm)	V-H	4 × 10	116
	• Lateral jumps over a bench (20 cm)	V-L	4 × 10	
	• Jumps with feet together and then separated throughout hoops	V-A-L	4 × 9	
7	• Jumps forward between barriers (30 cm)	V-H	4 × 12	120
	• Lateral jumps over a bench (20 cm)	V-L	4 × 9	
	• Jumps with feet together and then separated throughout hoops	V-A-L	4 × 9	
8	• Jumps forward between barriers (30 cm)	V-A	4 × 12	124
	• Lateral jumps over a bench (20 cm)	V-L	4 × 10	
	• Jumps with feet together and then separated throughout hoops	V-A-L	4 × 9	

*V-A, vertical-anterior-posterior; V-L, vertical-lateral; V-A-L, vertical-anterior-posterior-lateral.*

The MPT was always supervised by the same coach, and there were no injuries resulting from the training sessions during the intervention.

### Statistical Analysis

Statistical analyses were carried out using the SPSS 20 program for Windows (SPSS^®^, Chicago, IL, United States). Before using parametric statistics, the normality of data was confirmed, using the Shapiro-Wilk test. The sphericity was checked by the Mauchly test and, when it was not met, the significance of F-ratios was adjusted according to the Greenhouse-Geisser procedure or the Huynh-Feldt procedure. The test-retest reliability of measures was assessed using intra-class correlation coefficients (ICCs) (Vincent, 1995). Descriptive values are presented as means and standard deviations. The effects of the intervention were assessed using a 2-way analysis of variance with repeated measures: (EG vs. CG) × (before vs. after). To evaluate within-group pre-to-post performance changes, paired sample *t*-tests were applied. Effect sizes (ES) were determined by converting partial eta-squared values to Cohen’s d with the Excel spreadsheets. According to Cohen (1988) ES can be classified as small (0.00 ≤ *d* ≤ 0.49), medium (0.50 ≤ *d* ≤ 0.79), or large (*d* ≥ 0.80). The alpha level of significance was set at *p* < 0.05.

## Results

During our experiment, control group participants were trained for 23.64 ± 0.85 soccer training session and the experimental group for 23.57 ± 0.75 soccer training session and 15.71 ± 0.61 PT session. Furthermore, no significant inter-group difference was noted for anthropometric data at baseline (Table 1).

### SJ, CMJ, and *T*-Test Performance

Descriptive values of Pre and Post tests for SJ, CMJ and *T*-Test are presented in Table 4. ANOVA demonstrated significant group × time interaction for SJ, CMJ, and *T*-Test (*p* < 0.05 for all). For SJ, paired *t*-test demonstrated significant progress for EG however, no significant progress for CG (EG: *p* < 0.05, Δ = 11.14%; CG: *p* = 0.33, Δ = 0.42%). For CMJ, paired *t*-test demonstrated significant progress for EG however, no significant progress for CG (EG: *p* < 0.05, Δ = 9.91%; CG: *p* = 0.10, Δ = 0.42%). For *T*-test, paired *t*-test demonstrated significant progress for EG however, no significant progress for CG (*p* < 0.05, Δ = −3.07%; CG: *p* = 0.19, Δ = 0.42%).

**TABLE 4:** Vertical jump and *T*-Test performance before and after the intervention program.

**Group**	**Control**			**Paired *t*-test**	**Experimental**			**Paired *t*-test**	**ANOVA Group × Time**	
					
**Test**	**Pre**	**Post**	**% Δ**	***p* value**	**Pre**	**Post**	**% Δ**	***p* value**	***p* value**	**Cohen’s *d***
SJ (m)	0.19 ± 0.01	0.19 ± 0.01	0.42 ± 1.3	0.33	0.19 ± 0.02	0.21 ± 0.02	11.14 ± 2.9	0.00^∗^	0.00^∗^	5.77
CMJ (m)	0.21 ± 0.01	0.21 ± 0.02	−0.79 ± 1.8	0.10	0.21 ± 0.02	0.23 ± 0.02	9.91 ± 2.8	0.00^∗^	0.00^∗^	5.15
*T*-Test (s)	13.6 ± 0.7	13.6 ± 0.6	0.3 ± 0.7	0.19	13.7 ± 0.8	13.3 ± 0.8	−3.07 ± 1.1	0.00^∗^	0.00^∗^	3.53

*SJ, squat jump; CMJ, counter movement jump; ^∗^<0.05.*

### DPC on the Dominant Leg Performance

Descriptive values of Pre and Post tests of DPC on the dominant leg performance are presented in Table 5. ANOVA demonstrated significant group × time interaction for seven axes (anterior, lateral, postero-lateral, posterior, postero-medial, medial and antero-medial; *p* < 0.05 for all). However, no significant group × time interaction for antero-lateral axis (*p* = 0.50). Paired *t*-test demonstrated significant progress in EG, however, no significant change in CG: anterior (EG: *p* < 0.05, Δ = 3.79%; CG:*p* = 0.43, Δ = −0.26%); lateral (EG: *p* < 0.05, Δ = 2.18%; CG: *p* < 0.05, Δ = −0.92%); postero-lateral (EG: *p* < 0.05, Δ = 4.93%; CG:*p* = 0.08, Δ = −0.59%); posterior (EG: *p* < 0.05, Δ = 10.19%; CG:*p* = 0.76, Δ = −0.11%); postero-medial (EG: *p* < 0.05, Δ = 6.39%; CG: *p* = 0.85, **Δ = ** 0.07%); medial (EG: *p* < 0.05, Δ = 5.93%; CG: *p* = 0.11, **Δ = ** −0.51%); antero-medial (EG: *p* < 0.05, Δ = −3.79%; CG:*p* = 0.43, Δ = −0.26%).

**TABLE 5:** Dynamic postural control (SEBT) of the dominant leg performance before and after the intervention program.

**Group**	**Control**			**Paired *t*-test**	**Experimental**			**Paired *t*-test**	**ANOVA Group × Time**	
					
**Axe**	**Pre**	**Post**	**% Δ**	***p* value**	**Pre**	**Post**	**% Δ**	***p* value**	***p* value**	**Cohen’s *d***
Anterior	83.1 ± 1.7	83.3 ± 1.9	0.26 ± 1.2	0.43	80.9 ± 3.6	83.9 ± 3.7	3.79 ± 2.4	0.00^∗^	0.00^∗^	1.94
Antero-lateral	89.9 ± 2.3	89.2 ± 3.0	−0.8 ± 0.7	0.02^∗^	88.3 ± 4.4	88.8 ± 3.7	0.70 ± 2.3	0.32	0.50	0.19
Lateral	89.5 ± 2.6	88.7 ± 2.3	−0.92 ± 1.4	0.02^∗^	88.8 ± 3.6	90.7 ± 2.7	2.18 ± 2.1	0.00^∗^	0.00^∗^	1.76
Postero-lateral	84.4 ± 8.8	83.9 ± 8.6	−0.59 ± 1.1	0.08	82.3 ± 7.1	86.2 ± 5.4	4.93 ± 3.8	0.00^∗^	0.00^∗^	2.13
Posterior	67.7 ± 7.9	67.6 ± 7.6	−0.11 ± 2.1	0.76	65.0 ± 5.9	71.4 ± 5.3	10.19 ± 6.2	0.00^∗^	0.00^∗^	2.43
Postero-medial	77.6 ± 4.1	77.7 ± 4.4	0.07 ± 1.8	0.85	74.9 ± 4.2	79.6 ± 3.9	6.39 ± 3.1	0.00^∗^	0.00^∗^	2.54
Medial	77.1 ± 3.8	76.6 ± 3.8	−0.51 ± 1.1	0.11	74.0 ± 5.0	78.4 ± 5.1	5.93 ± 2.8	0.00^∗^	0.00^∗^	3.21
Antero-medial	82.6 ± 4.5	81.8 ± 4.8	−0.97 ± 1.4	0.02^∗^	79.2 ± 4.8	82.5 ± 5.0	4.08 ± 2.1	0.00^∗^	0.00^∗^	2.93

*^∗^<0.05.*

### DPC on the Non-dominant Leg Performance

Descriptive values of Pre and Post tests of DPC on the non-dominant leg performance are presented in Table 6. ANOVA demonstrated significant group × time interaction for seven axes (anterior, antero-lateral, lateral, posterior, postero-medial, medial and antero-medial; *p* < 0.05 for all). However, no significant group × time interaction for postero-lateral axis (*p* = 0.54). Paired *t*-test demonstrated significant progress in EG, however, no significant change in CG: anterior (EG: *p* < 0.05, Δ = 7.25%; CG: *p* = −0.12, Δ = −0.96%); antero-lateral (EG: *p* < 0.05, Δ = 2.80%; CG:*p* = 0.07, Δ = −0.58%); lateral (EG: *p* < 0.05, Δ = 1.17%; CG: *p* = 0.08, Δ = 0.76%); posterior (EG: *p* < 0.05, Δ = 4.96%; CG:*p* = 0.85, Δ = −0.04%); postero-medial (EG: *p* < 0.05, Δ = 3.89%; CG: *p* < 0.05, Δ = −0.98%); medial (EG: *p* < 0.05, Δ = 5.89%; CG: *p* < 0.05, Δ = −0.05%); antero-medial (EG: *p* < 0.05, Δ = 4.86%; CG:*p* = 0.43, Δ = −1.21%).

**TABLE 6:** Dynamic postural control (SEBT) of the non-dominant leg performance before and after intervention program.

**Group**	**Control**			**Paired *t*-test**	**Experimental**			**Paired *t*-test**	**ANOVA Group × Time**	
					
**Axe**	**Pre**	**Post**	**% Δ**	***p* value**	**Pre**	**Post**	**% Δ**	***p* value**	***p* value**	**Cohen’s *d***
Anterior	67.7 ± 4.1	67.0 ± 4.4	−0.96 ± 2.1	0.12	65.5 ± 4.2	70.2 ± 3.6	7.25 ± 4.2	0.00^∗^	0.00^∗^	2.70
Antero-lateral	87.3 ± 3.1	86.8 ± 2.8	−0.58 ± 1.1	0.07	86.2 ± 4.4	88.6 ± 4.3	2.80 ± 2.3	0.00^∗^	0.00^∗^	1.95
Lateral	89.5 ± 2.3	90.5 ± 2.7	0.76 ± 1,2	0.08	91.6 ± 3.1	92.6 ± 2.8	1.17 ± 1.8	0.04^∗^	0.00^∗^	0.10
Postero-lateral	91.0 ± 2.9	89.0 ± 4.9	−2.23 ± 4.3	0.07	89.5 ± 6.5	89.0 ± 7.6	−0.14 ± 9.4	0.84	0.54	0.23
Posterior	80.0 ± 4.0	80.1 ± 4.3	0.04 ± 1.2	0.85	77.0 ± 5.1	80.7 ± 4.1	4.96 ± 3.3	0.00^∗^	0.00^∗^	2.04
Postero-medial	82.5 ± 2.3	81.6 ± 2.6	−0.98 ± 1.6	0.04^∗^	80.8 ± 3.1	84.0 ± 2.3	3.89 ± 2.0	0.00^∗^	0.00^∗^	2.86
Medial	74.0 ± 3.1	73.2 ± 3.3	−1.05 ± 0.9	0.00^∗^	73.3 ± 4.0	77.6 ± 4.3	5.89 ± 2.5	0.00^∗^	0.00^∗^	3.76
Antero-medial	75.6 ± 4.2	74.6 ± 4.0	−1.21 ± 1.8	0.03^∗^	74.2 ± 5.6	77.8 ± 5.6	4.86 ± 2.4	0.00^∗^	0.00^∗^	2.96

*^∗^<0.05.*

## Discussion

The main objective of the present study was to investigate the effects of low volume MPT on the vertical jump performance, CODP, and DPC in young soccer players. The result shows that the MPT enhanced three important qualities that are relevant to the performance of young male soccer players (i.e., vertical jump height, CODP, and DPC).

Previously to the present work, several studies have examined the effects of unidimensional PT in young male soccer players; these have used high volume programs lasting from 8 to 16 weeks (Diallo et al., 2001; Meylan and Malatesta, 2009; Sohnlein et al., 2014) with a potential risk of overuse injuries in the growing athletes who could potentially undergo growth related injuries. Other researchers (Ramirez-Campillo et al., 2014; Chaabene and Negra, 2017) interestingly proposed lower volume programs and still found significant gains in factors related to athletic performance. Moreover, Chaabene and Negra (2017) showed no significant difference of adaptations between high and low volume plyometrics programs in young soccer players, perhaps because the low volume training stimulus already elicits an optimal adaptive response in this population. In accordance with the findings of these studies, the present study was based on progressive and moderate intensity exercise in order to minimize the risk of overuse injuries in the young participants. Moreover, the singularity of the present study (compared to previous literature) is that it is unique by the combination of actions in the multidirectional planes to better meet the multi-directional needs of soccer activity.

Plyometric training (PT) improves exercise performance that involves SSC of muscle–tendon units (Markovic and Mikulic, 2010). Hirayama et al. (2017) demonstrated that PT improved the SSC exercise performance by the optimization of muscle-tendon behavior of the agonists, associated with an alteration in the neuromuscular activity during SSC exercise and an increase in tendon stiffness. Furthermore, a decrease in the neuromuscular activity of the antagonists during the braking phase appears to play an important role in this improvement. These findings can explain why the present study showed a significant improvement (*p* < 0.05) in both types of vertical jumps with two different regimes [concentric regime for the squat jump (SJ, Δ = 11.14%) and plyometric regime for the counter movement jump (CMJ, Δ = 9.91%)]. But, it is important to point out that the characteristics of the muscle-tendon unit are different between youth and adults. Indeed, Mersmann et al. (2014) showed imbalanced development of muscle strength and tendon mechanical and morphological properties in adolescent athletes compared to middle-aged athletes. According to these specific characteristics in young people, the effect of a plyometric program on the SSC may be different compared to adults. But until now, to the best of the authors’ knowledge, there has been no study on this topic in young athletes.

MPT induced significant improvements in vertical jump height, possibly reflecting increased strength, and/or power of the leg extensor muscles (Michailidis et al., 2013), better coordination of agonists and antagonists, and/or a greater recruitment of motor units (Garcia-Pinillos et al., 2014). One weakness of the present study is that we did not measure strength itself, but have assessed proxies to strength (from vertical jumping height). Indeed, vertical jump height (during SJ and CMJ) is strongly correlated with the lower limbs’ strength in young soccer players (Chamari et al., 2004). This suggests that the significant improvement in vertical jump height reflects a possible significant improvement in lower limb strength in the EG.

MPT also improved performance of the COD, corroborating the earlier findings of Miller et al. (2006) following 6 weeks of in-season PT. Such gains probably reflect increases in muscular power and movement efficiency (Miller et al., 2006), qualities that are important in team sports.

Finally, the present program enhanced postural control in multiple axes and inversely with the control group not performing MPT, the performance of DPC on several axes decreased. This suggests that when there is no specific preparation program to improve DPC in the sports season, this quality declines and consequently the risk of injury might potentially increase. This improvement in postural control for the experimental group could reflect either improvements in motor output of the lower extremity muscles (Mirwald et al., 2002), and/or changes in proprioception and neuromuscular control (Hewett et al., 2002). Dynamic balance was improved in both anterior-posterior and medial-lateral directions, in contrast with the study of Ramirez-Campillo et al. (2015), where vertical and horizontal exercises improved dynamic balance in the anterior-posterior but not in the medial-lateral direction. Taken all together these results corroborate the fact that an increase in the lower-extremity muscle power would be associated with enhancements of the postural performance (Paillard, 2017a). This underlines the importance of the present study with its’ innovative multidirectional approach.

Although the present study points to the effectiveness of MPT, there remains a need to undertake a direct comparison between uni-, bi- and multi-directional plyometrics programs. Despite their importance in soccer, the present study did not include any goal keeper. Indeed, the latter players’ training regimens and physical capacities are markedly different compared to outfield players. Therefore, (i) in order to avoid having eventual outliers in our study population and (ii) for practical reasons of training organization on the field, we have chosen to exclude them from the study. Further studies on goal keepers are therefore warranted.

## Practical Applications

The findings of the present study have important practical applications. It was shown for the first time that the MPT enhanced three important qualities that are relevant to the performance of young male soccer players (vertical jump height, CODP, and DPC). Such a program has the potential to be very much appealing to coaches as requiring little execution time while yielding valuable and relevant outcomes in the physical preparation of young soccer players.

## Data Availability Statement

The datasets generated for this study are available on request to the corresponding author.

## Ethics Statement

The studies involving human participants were reviewed and approved by the Manouba University Institutional Review Committee for the ethical use of human participants. Written informed consent to participate in this study was provided by the participants’ legal guardian/next of kind.

## Author Contributions

MJ and GR performed the experiment and collected the data. All authors contributed to the study design, data analysis, and writing the manuscript.

## Conflict of Interest

The authors declare that the research was conducted in the absence of any commercial or financial relationships that could be construed as a potential conflict of interest.
